# Quantitative scoring of epithelial and mesenchymal qualities of cancer cells using machine learning and quantitative phase imaging

**DOI:** 10.1117/1.JBO.25.2.026002

**Published:** 2020-02-18

**Authors:** Van K. Lam, Thanh Nguyen, Vy Bui, Byung Min Chung, Lin-Ching Chang, George Nehmetallah, Christopher B. Raub

**Affiliations:** a The Catholic University of America, Department of Biomedical Engineering, Washington, DC, United States; b The Catholic University of America, Department of Electrical Engineering and Computer Science, Washington, DC, United States; c The Catholic University of America, Department of Biology, Washington, DC, United States

**Keywords:** holography, quantitative phase, machine learning, epithelial, mesenchymal, cancer cells, support vector machine

## Abstract

**Significance**: We introduce an application of machine learning trained on optical phase features of epithelial and mesenchymal cells to grade cancer cells’ morphologies, relevant to evaluation of cancer phenotype in screening assays and clinical biopsies.

**Aim**: Our objective was to determine quantitative epithelial and mesenchymal qualities of breast cancer cells through an unbiased, generalizable, and linear score covering the range of observed morphologies.

**Approach**: Digital holographic microscopy was used to generate phase height maps of noncancerous epithelial (Gie-No3B11) and fibroblast (human gingival) cell lines, as well as MDA-MB-231 and MCF-7 breast cancer cell lines. Several machine learning algorithms were evaluated as binary classifiers of the noncancerous cells that graded the cancer cells by transfer learning.

**Results**: Epithelial and mesenchymal cells were classified with 96% to 100% accuracy. Breast cancer cells had scores in between the noncancer scores, indicating both epithelial and mesenchymal morphological qualities. The MCF-7 cells skewed toward epithelial scores, while MDA-MB-231 cells skewed toward mesenchymal scores. Linear support vector machines (SVMs) produced the most distinct score distributions for each cell line.

**Conclusions**: The proposed epithelial–mesenchymal score, derived from linear SVM learning, is a sensitive and quantitative approach for detecting epithelial and mesenchymal characteristics of unknown cells based on well-characterized cell lines. We establish a framework for rapid and accurate morphological evaluation of single cells and subtle phenotypic shifts in imaged cell populations.

## 1 Introduction

Quantitative phase imaging (QPI) is a label-free optical imaging technique that measures the phase delay introduced when a coherent laser beam travels through a thin transparent specimen, such as cells.^1^ The uncorrected optical pathlength yields information about cell morphology and geometric thickness of the specimen along its phase projection, as well as fluctuations in the local index of refraction.^2^ Reconstructed optical phase maps that result from QPI of cells possess phase texture in the variation of pixel values within the boundary of the cell,^3^ quantified through texture parameters.^4^ The pixel information from phase maps is relevant to cell structure and function. For example, a cell’s refractive index can reveal its total protein concentration,^5^ organization, and distribution of subcellular organelles, which are distinctive for certain biological phenotypes.^6^ Hence, a collection of quantitative parameters from optical phase maps constitutes a cell type’s phase signature, providing additional information beyond the cell shape.

Digital holographic microscopy (DHM) is one type of QPI that uses the holography principle to determine the amplitude and phase of the diffraction wavefront^7^^,^^8^ and is well-suited for imaging biological specimens. Telecentric DHM is able to optically compensate for phase aberrations introduced by an imaging objective, making the computational removal of additional optical aberrations easier.^2^ Other key features of DHM include noninvasive imaging with low power density at the specimen, high temporal resolution, and rich quantitative pixel information. Recent applications of DHM to the assessment of living biological specimens include characterization of the global morphology of confluent cell layers,^9^ analysis of cell proliferation and morphology on various substrates,^1^^,^^3^^,^^10^ cell responses to drugs,^11^^,^^12^ determination of phase features relating to cell motility,^4^^,^^13^ and cell classification in flow cytometry.^14^^,^^15^

Machine learning applications to QPI include rapid evaluation and classification of cell types and (patho)physiological states^4^^,^^14^^–^^20^ and improvements in reconstructed image quality.^21^^,^^22^ Phase texture from pixels, shape features of adherent cells,^4^ and dry mass/volumetric determination of cells in liquid suspension^15^ are quantitative parameters that served as predictors for binary classification of cancer and noncancerous cells. Support vector machines (SVMs) have been particularly successful in classification of cell lines using such quantitative phase parameters.^4^^,^^15^^,^^23^^,^^24^ Ensemble methods train multiple weak learners and then combine them to obtain better predictive performance for classification. For example, the Boosting ensemble method trains learners sequentially, focusing subsequent models on the previous models’ miss-classifications, while the Bagging ensemble method trains learners independently. A recent study suggested that ensemble methods could improve detection of clear cell renal cell carcinoma in kidney disease leading to improved diagnosis and treatment.^25^

QPI has great potential to evaluate cells in thin sections and in cell-based screening assays. For example, machine learning classification from QPI compared favorably to manual scoring of the Gleason grade of prostate cancer from histology sections ^26^ or to conventional screening in terms of predicting pathological features of hematological diseases.^27^ The use of parameters from QPI has recently been explored to assess shifts in population distributions of cell phase parameters, indicating altered phenotype, or to differentiate multiple bacteria species based on their single-cell profiling capability.^28^^,^^29^ The effects of cell seeding density,^4^ exposure to anticancer drugs,^30^^,^^31^ and other influences on cell phenotype^32^^–^^34^ have been robustly evaluated with QPI. Quantitative imaging and machine learning have the potential to save time, labor, and reduce human error in phenotypic profiling, which could help pathologists and scientists to accurately detect circulating tumor cells,^35^ classify cancer cells,^36^^,^^37^ evaluate the metastatic potential of cancer cells,^38^ and assess cancer drug resistance.^39^ Thus, machine learning-assisted QPI has great power to aid in interpreting large-scale and high-dimensionality data from cells, potentially enhancing cancer diagnosis and treatment.

A key aspect of cancer relevant to disease outcomes is cancer cell morphology. Many cancers adopt either “epithelial” or “mesenchymal” morphologies, dependent on certain gene mutations, gene expression profiles influenced by the microenvironment, and epigenetic changes.^40^ Indeed, the route to transformation for many precancers involves epithelial-to-mesenchymal transition in which cells switch from a quiescent phenotype with rounded morphology to an actively motile, invasive phenotype with elongated morphology.^41^^,^^42^ Complicating this picture, some cancer cell lines, such as MCF-7 cells, are rounded and form aggregates *in vitro* and yet are more invasive than cancer cell lines with single, elongated cell morphologies.^43^ Another well-studied breast cancer cell line, MDA-MB-231, adopts elongated, mesenchymal, and rounded amoeboid morphologies as a bimodal invasion strategy to overcome microenvironmental barriers.^44^ In previous studies, SVMs were used to classify rounded and elongated MDA-MB-231 cells^3^ and distinguish MCF-7 and MDA-MB-231 cells from noncancerous epithelial and mesenchymal cell lines.^4^ These studies raised the question of whether a universal score could be developed to grade cells along the spectrum of epithelial to mesenchymal features.

Since results from previous studies classified cells based on textural and shape-based phase map features, we hypothesized that a quantitative score from machine learning algorithms trained on noncancerous epithelial and mesenchymal cell lines could be used to assign mesenchymal or epithelial morphological status to cancer cells. To test this hypothesis, a binary classifier of two noncancerous gingival cell lines, one epithelial and one fibroblast/mesenchymal, was evaluated. Then the algorithm trained on noncancerous cells was applied to two cancer cell lines of mixed morphology and an “epithelial–mesenchymal” (EM) score was derived. Results indicate that such an approach accurately classifies epithelial and mesenchymal cell lines and assigns cancer cells a phenotypic score on the EM axis consistent with observed morphology. We propose this approach of deriving morphological phenotypic scores from machine learning on archetypal cells as a generally useful and robust way to assess phenotypic characteristics of unknown cell populations and single cells, which holds promise for future clinical and research applications.

## 2 Materials and Methods

### 2.1 Cell Culture

Cell culture procedures were the same as in Ref. 4. For DHM imaging, cells were passaged when reaching 80% to 90% confluence and seeded on glass-bottomed Petri dishes. Immortalized human gingival keratinocytes (Gie-No3B11, abbreviated as GIE, derived from buccal gingiva),^45^ immortalized human gingival fibroblasts (HGF, derived from American Type Culture Collection CRL-2014 primary gingival cells),^46^^,^^47^ and the breast cancer cell lines MCF-7^48^ and MDA-MB-231,^49^ both adenocarcinomas derived from pleural effusions, were seeded at respective densities of 60,000; 40,000; 40,000; and 30,000 cells in a 35-mm-diameter glass-bottomed Petri dish (Part #229632, CELLTREAT Scientific Products, Pepperell, Massachusetts). The different densities were estimated to produce a roughly equal number of cells per field of view after 24 h due to differences in growth rates and aggregation. Cancer cell lines were fed with Dulbecco’s modified Eagle’s medium (Lot # SLBW4140, Sigma-Aldrich, St. Louis, Missouri), supplemented with 10% Fetalgro (Rocky Mountain Biologicals, Missoula, Montana) and 1% penicillin-streptomycin (Corning Inc., Corning, New York). The HGF and GIE cell lines were cultured in Prigrow 3 and Prigrow 4, respectively (Applied Biological Materials, Inc., British Columbia, Canada). Nutrient media for gingival cell lines were supplemented with 10% fetal bovine serum and 1% penicillin-streptomycin. Cells adherent after 24 h were fed with 200  μl of fresh, prewarmed media and were covered with sterile cover slips. To avoid effects on cells from the ambient environment, each imaging session was performed over 15 to 20 min of total time out of the incubator.

### 2.2 Digital Holographic Microscopy Setup, Imaging, and Preprocessing

**Fig. 1:**
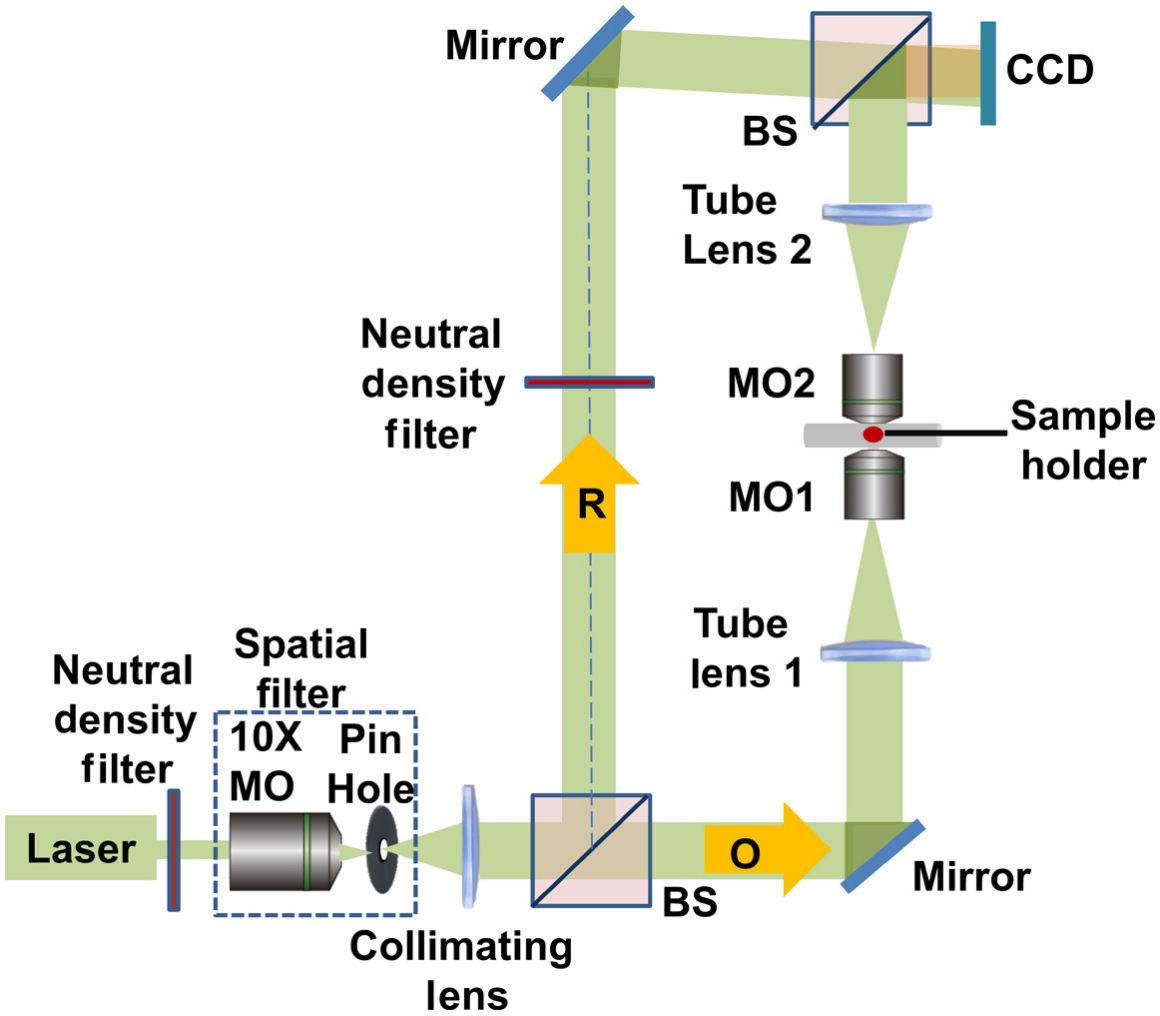
The bitelecentric DHM in transmission configuration, including MO, beamsplitters (BS), object beam (O), reference beam (R), and CMOS camera.

A detailed description of the telecentric DHM setup and image processing to optically compensate for phase aberrations is described in previously published studies.^2^^,^^3^^,^^50^ The telecentric DHM setup (Fig. 1) is based on a bitelecentric configuration that optically cancels the bulk of the spherical aberrations caused by the microscope objectives (MOs).^51^^–^^53^ The lateral resolution was 1.2  μm with $0.18\times 0.18\;\mu m^{2}\text{ pixel}$ dimensions of the lateral reconstruction. A 632-nm-wavelength He-Ne laser was used to generate sample and reference beams that recombined at the camera sensor plane as holograms. The holograms were captured by a 1.3-MP CMOS camera (Lumenera Corporation, Inc., Ontario, Canada) and the reconstructed phase map was obtained using the Fresnel reconstruction algorithm.^2^^,^^54^

Principal component analysis (PCA) was employed to cancel the main hologram phase aberrations. The following steps summarize the PCA algorithm: (1) perform singular value decomposition to obtain the first dominant principal component (PC), (2) obtain the linear and quadratic coefficients of the phase vectors from least square fitting of the two dominant singular vectors, (3) use these coefficients to compute the phase at the camera sensor, φ(k,l), and (4) multiply the conjugate φ*(k,l) with the hologram to obtain $\exp[-j\varphi_{\mathrm{ob}}(k,l)]$, which is the phase due to the biological sample without contributions of MOs and tilt. Phase height was determined from the reconstructed optical pathlength by dividing by an assumed average index of refraction mismatch between cells and surrounding media of Δn=1.381−1.337=0.044.

### 2.3 Machine Learning and Epithelial–Mesenchymal Score Generation

**Fig. 2:**
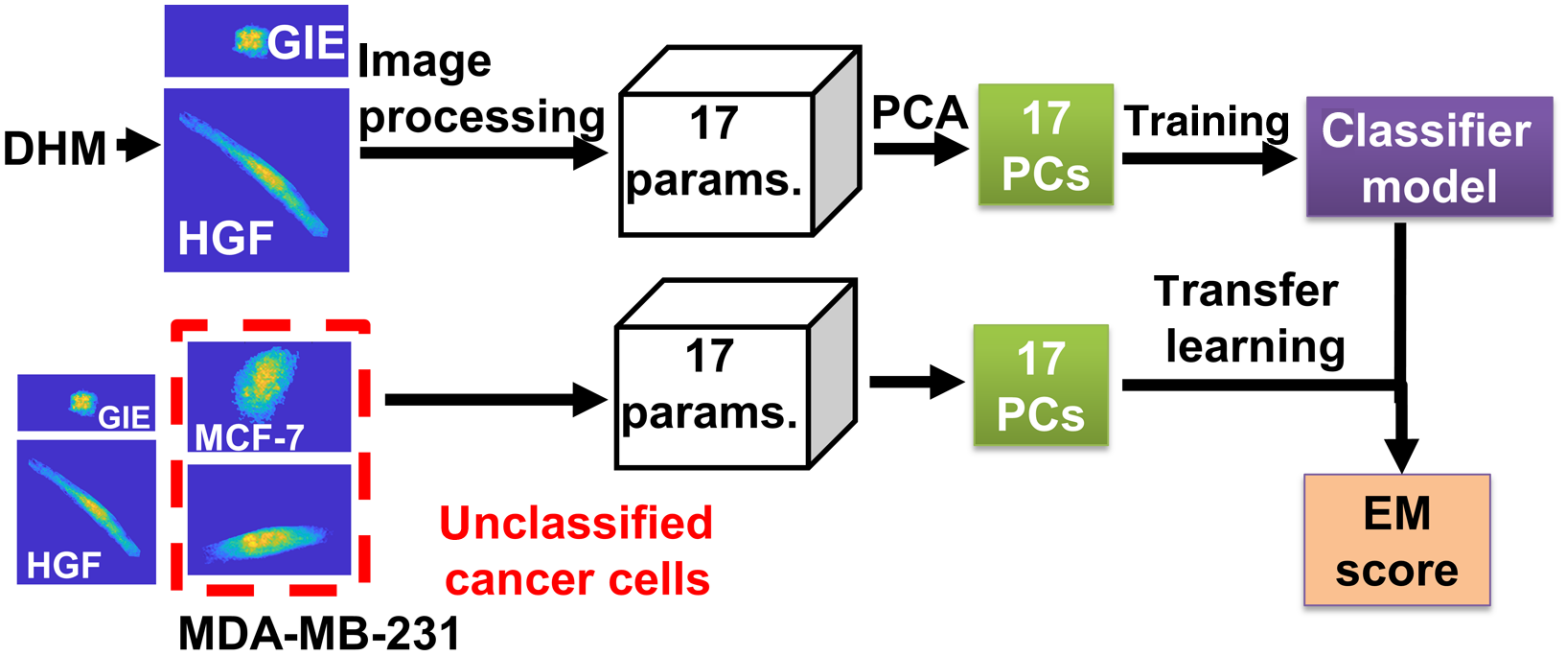
Machine learning was performed on 1 to 17 features derived from phase maps reconstructed from adherent cells’ holograms. Derived features from epithelial (GIE) and mesenchymal (HGF) cell types were used for training. For transfer learning, six PCs representing most of the variation in cell phase maps from two untrained cancer cell lines, MCF-7 and MDA-MB-231, were used for testing and to generate machine learning prediction scores as candidates for an EM score.

Machine learning algorithms were evaluated and used to classify gingival cells and for transfer learning on cancer cells to define an EM score (Fig. 2). Cells were segmented and 17 phase parameters were extracted from each of the four cell lines using a custom-written code in MATLAB (version R2015a), which was described previously.^34^ Parameters are described in Table S1 in the Supplementary material. In total, there were 1295 cells from four different cell lines, which were segmented throughout this study, including 332 cells of GIE, 309 cells of HGF, 307 cells of MCF-7, and 347 cells of MDA-MB-231.

Data were randomly partitioned at a ratio of 4:1 for training and testing. Training was performed on parameters from 252 and 229 GIE and HGF cells, respectively, following a fivefold cross validation. First, the 17 phase parameter predictors were transformed into PCs using PCA, and the PCA-transformed data were used as inputs for training following fivefold cross validation. Training and cross validation using linear SVM were performed five times each, selecting 1 to 17 PCs as predictors. To evaluate the highest prediction accuracy during training, a one-factor repeated measures analysis of variance (ANOVA) was performed for accuracy on training performance resulting in 1 to 17 PCs used, with a Dunnett’s *post-hoc* test to compare results to those of one PC. Then several single and ensemble methods were trained using the same number of PCs found to produce the highest accuracy from the linear SVM algorithm. Default settings in MATLAB were used for each classifier, including a cost parameter of 1 for misclassification. Accuracy was evaluated by comparing output labels to true cell line labels. These were compared to each other using a two-tailed Student’s t-test. Results were reported as the mean ± standard deviation. Plots of the first two PCs and receiver operating characteristic (ROC) curves for the best single and best ensemble method classifiers were constructed.

These most accurate single and ensemble algorithms in training were exported as two models in the MATLAB workspace using the classification Learner application. Each model was applied to the PCA-transformed data of 307 cells from MCF-7 and 347 cells from MDA-MB-231 combined with the 80 cells of GIE and 80 cells of the HGF cell lines used for testing. In addition to the classification accuracy, SVM scores, Boosted Trees (AdaBoost) scores, Bagged Trees scores, and SVM posterior probabilities, defined below, were also calculated. Cells from the two cancer cell lines MDA-MB-231 and MCF-7 were assigned as either mesenchymal or epithelial based on the binary classifier. All scores and posterior probabilities were plotted in histograms to evaluate the performance as an EM score. In addition, SVM scores and posterior probabilities were correlated to determine the relative sensitivity of the score and probability throughout their respective ranges. The SVM score $s_{j}$, the distance of the observation j to the decision boundary, was calculated as^55^

$$s_{j}={\left(\frac{x_{j}}{s_{k}}\right)}^{\prime }\beta +b,$$ (1)

where $x_{j}$ is the predictor data of observation j, $s_{k}=2.5196$ is the linear kernel scale, β is the vector of fitted linear coefficients, and b is the intercept of the hyperplane defining the separation. The posterior probability $P(s_{j})$ was calculated as^56^

$$P(s_{j})=1/(1+\exp(As_{j}+B)),$$ (2)

where A and B are the fitted slope and intercept, respectively, of the sigmoid function. Meanwhile, the prediction score for AdaBoost, ranging from −∞ to +∞, was defined as^57^

$$f(x)=\overset{T}{\underset{t=1}{\sum }}[a_{t}h_{t}(x)],$$ (3)

where $a_{t}=0.5\;\log[(1-\varepsilon_{t})/\varepsilon_{t}]$ are the weights of the sequential learners’ hypotheses, $\varepsilon_{t}$ is the weighted classification error of learner t, and $h_{t}(x)$ is the prediction of learner t for prediction data x for T total learners. The prediction scores for Boosted Trees are estimated posterior probabilities:^58^

P^bag(c|x)=∑t=1T[atP^t(c|x)I(t∈S)]/∑t=1T[atI(t∈S)], (4)

where P^t(c|x) is the estimated posterior probability of learner t for class c with given predictor data x, and I(t∈S) is 1 when learner t is of the indices S from trees used in the prediction, otherwise it is 0.

## 3 Results

### 3.1 Cell Morphologies from Optical Phase Maps Vary Across and Within Cell Lines

**Fig. 3:**
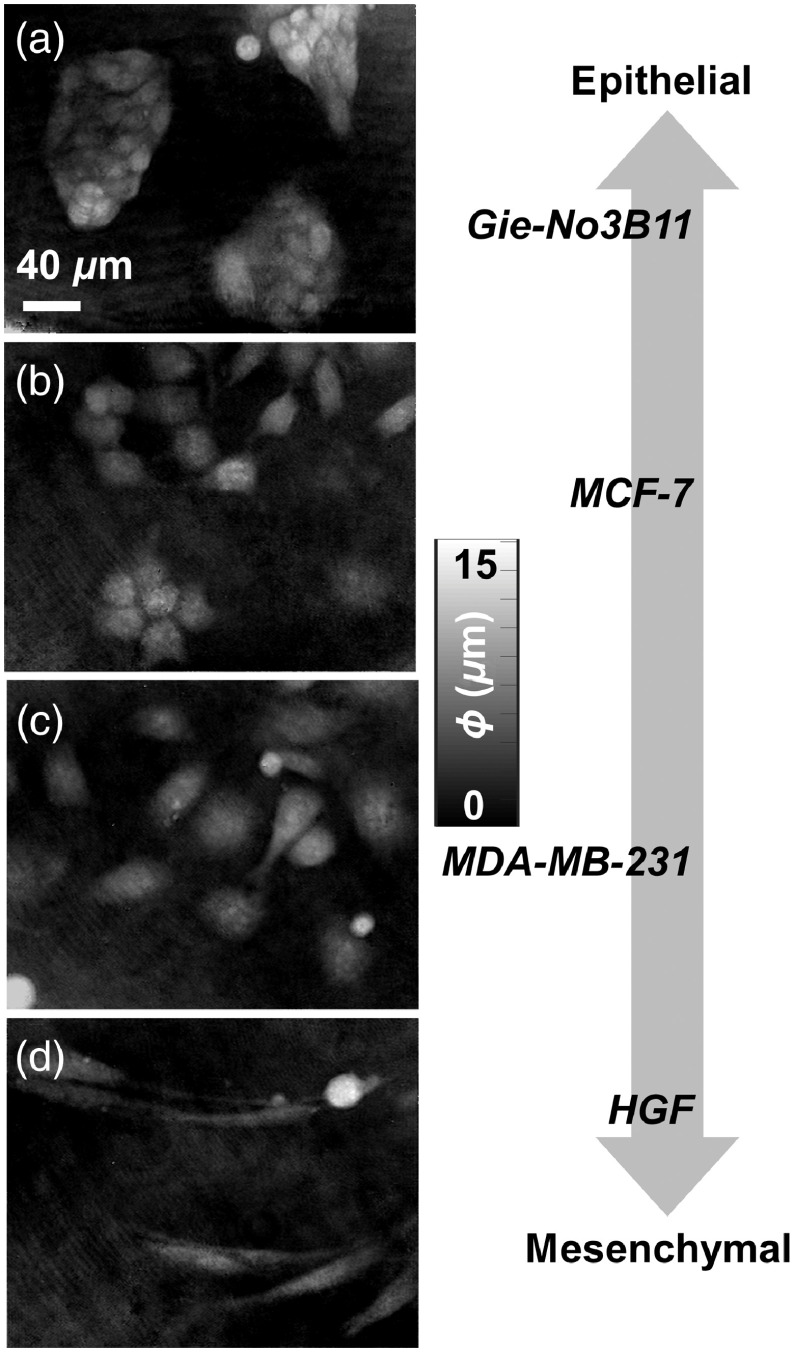
Representative DHM phase maps from living, adherent cells of (a) Gie-No3B11 (GIE), (b) MCF-7, (c) MDA-MB-231, and (d) HGF cell lines. The cells are ordered qualitatively on an EM axis based on cell morphology apparent in phase maps. Scale and phase bars are indicated.

Cell shapes from GIE [Fig. 3(a)] and HGF [Fig. 3(d)] cell lines resembled epithelial and mesenchymal morphologies, respectively. While GIE cells were more rounded and aggregated in clusters, HGF cells were more elongated with lower phase signals in pixels within the cell body. The cancer cell lines had morphologies in between GIE and HGF cells, with a more punctate phase texture [Figs. 3(b) and 3(c)]. Cells from the MCF-7 cell line form epithelial-like clusters with sharp cell and cluster boundaries [Fig. 3(b)]. Cells from the MDA-MB-231 cell line [Fig. 3(c)] appeared both rounded and elongated and were typically isolated.

### 3.2 Classification of Epithelial and Mesenchymal Cell Lines Is Highly Accurate

**Fig. 4:**
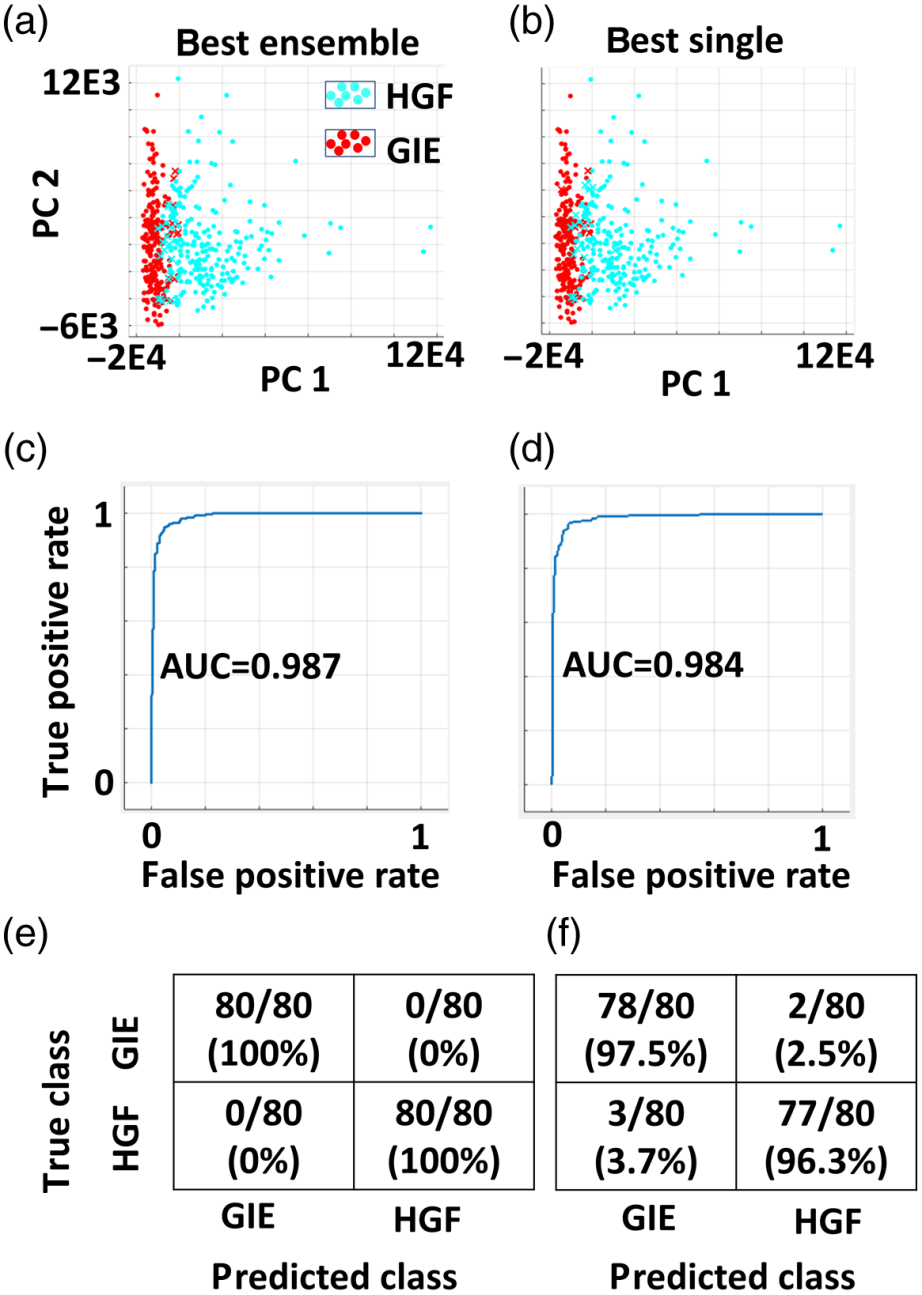
Binary classifier training data (a) and (b) scatterplots of PC 1 versus 2, highlighting correctly classified GIE (red circle) and HGF (cyan circle) cells, and misclassified cells (red and cyan x’s, with color representing the true class). (c) and (d) ROC curves from training data, with AUC listed. (e) and (f) Error tables from a test dataset for (e) Bagged Trees and (f) linear SVM.

Binary classification was evaluated for multiple algorithms available in the MATLAB machine learning and statistics toolbox, using the training set of 481 cell phase maps (n=252 from the GIE cell line; n=229 from the HGF cell line), all PCs as predictors, with accuracies ranging from 82% to 96% and the highest for linear SVM. Tuning the hyperparameters of box constraint level and kernel scale did not improve training accuracy. Therefore, the number of PCs used as predictors to linear SVM was varied from 1 to 17 (Table 1). Linear SVM with 6, 8, and 17 PCs all produced higher training accuracies than 1 PC (ANOVA, F=47.6, p<0.001, Dunnett’s test versus 1 PC, p<0.001). Six PCs were selected for use based on this statistical test and on previous models classifying cells based on phase features, which selected six PCs as the smallest number producing no increase in area under the curve (AUC) of ROC curves. Linear SVM training resulted in an accuracy of 95.5%±0.3%. Training using SVMs with different kernel functions (quadratic, cubic, Gaussian), decision trees, or k=1 nearest-neighbor methods did not improve accuracy. The best ensemble method classifier was Bagged Trees (Bag ensemble method, 200 learners, and learning rate of 0.1), which did not improve the accuracy more than the best single method (t-test, p=0.25). Boosted Trees (AdaBoost algorithm, 200 learners, 0.1 learning rate) produced lower accuracy than linear SVM, each trained on six PCs (t-test, p<0.01). Figures 4(a)–4(d) provide scatterplots of PCs 1 versus 2 and ROC curves for the best performing single and ensemble methods from model training and validation.

**Table 1:** Training accuracy of various machine learning algorithms to classify epithelial and mesenchymal cells.

Type	Method	Accuracy (%, μ±SD)
Single	Linear SVM, 1 PC	93.0±0.2
	Linear SVM, 2 PCs	93.0±0.2
	Linear SVM, 4 PCs	92.8±0.3
	Linear SVM, 5 PCs	93.4±0.6
	Linear SVM, 6 PCs*	95.5±0.3
	Linear SVM, 8 PCs*	95.1±0.3
	Linear SVM, 17 PCs*	95.2±0.6
	Other SVMs, 6 PCs	95.4±3.9
	Decision trees, 6 PCs	91.6±0.2
	Nearest neighbor, 6 PCs	88.9±3.7
Ensemble	Boosted Trees, 6 PCs, AdaBoost**	94.3±0.5
	Bagged Trees, 6 PCs, Bag	95.2±0.4

* p<0.001, ANOVA and *post-hoc* Dunnett’s test versus linear SVM, 1 PC.

** p<0.001, Student’s t-test versus linear SVM, 6 PCs.

**Fig. 5:**
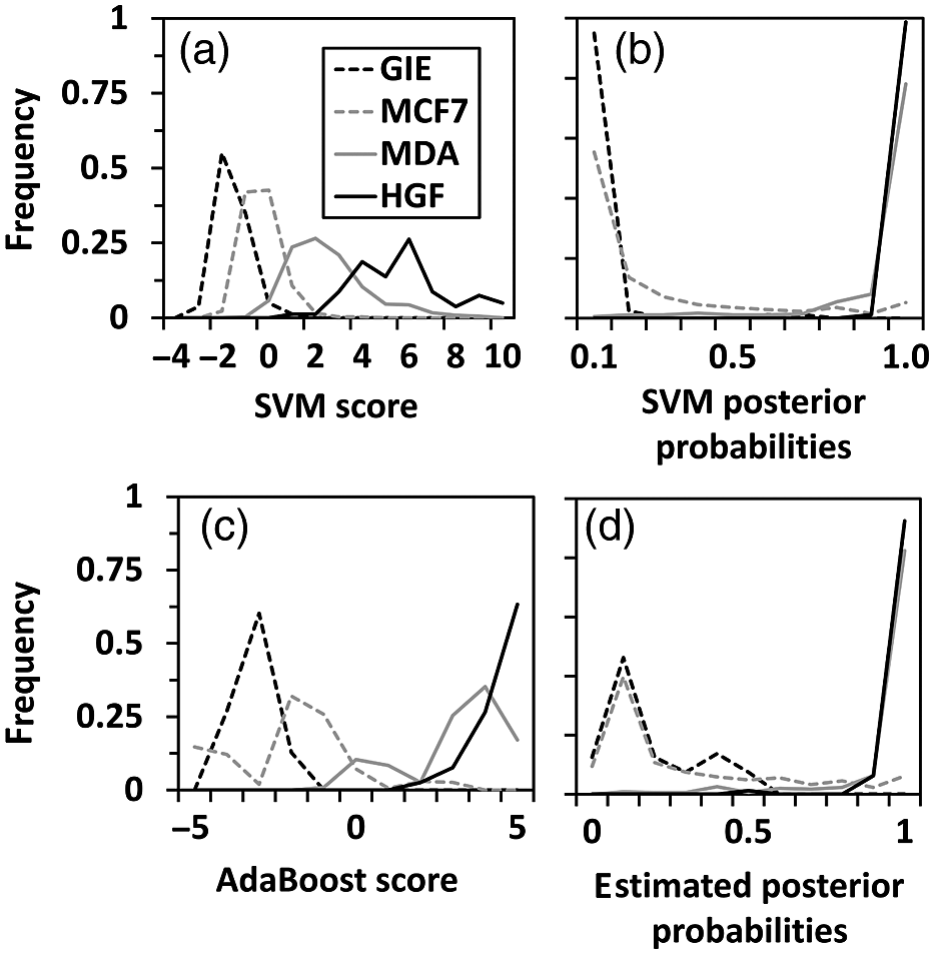
Prediction scores for transfer learning of an EM classifier to cancer cells. Histograms of prediction score distributions for (a) SVM predictions, (b) SVM posterior probabilities, (c) AdaBoost predictions, and (d) estimated posterior probabilities from Boosted Trees for test datasets of n=80 GIE cells (black dashed line) and n=80 HGF cells (black solid line), and transfer learning datasets of n=307 MCF-7 cells (gray dashed line) and n=347 MDA-MB-231 cells (gray solid line).

Testing based on the linear SVM and Bagged Trees models on a naive dataset of n=80 cells each from GIE and HGF cell lines produced error rates of 2.5% to 3.7% and 0%, respectively [Figs. 4(e), 4(f)]. Transfer learning using the linear SVM model classified 286/307 (87.0%) of MCF-7 cells as epithelial (GIE class) and 326/347 (93.9%) of MDA-MD-231 cells as mesenchymal (HGF class, data not shown). Transfer learning using the Bagged Trees model classified 262/307 (78.1%) of MCF-7 cells as epithelial (GIE class) and 329/347 (94.8%) of MDA-MD-231 cells as mesenchymal (HGF class, data not shown). Linear SVM, Bagged Trees, and Boosted Trees algorithms were used to calculate prediction scores for each cell of the test and transfer datasets (Fig. 5).

### 3.3 Binary Epithelial–Mesenchymal Classifier Prediction Scores Separate Cancer Cells by Morphology

**Fig. 6:**
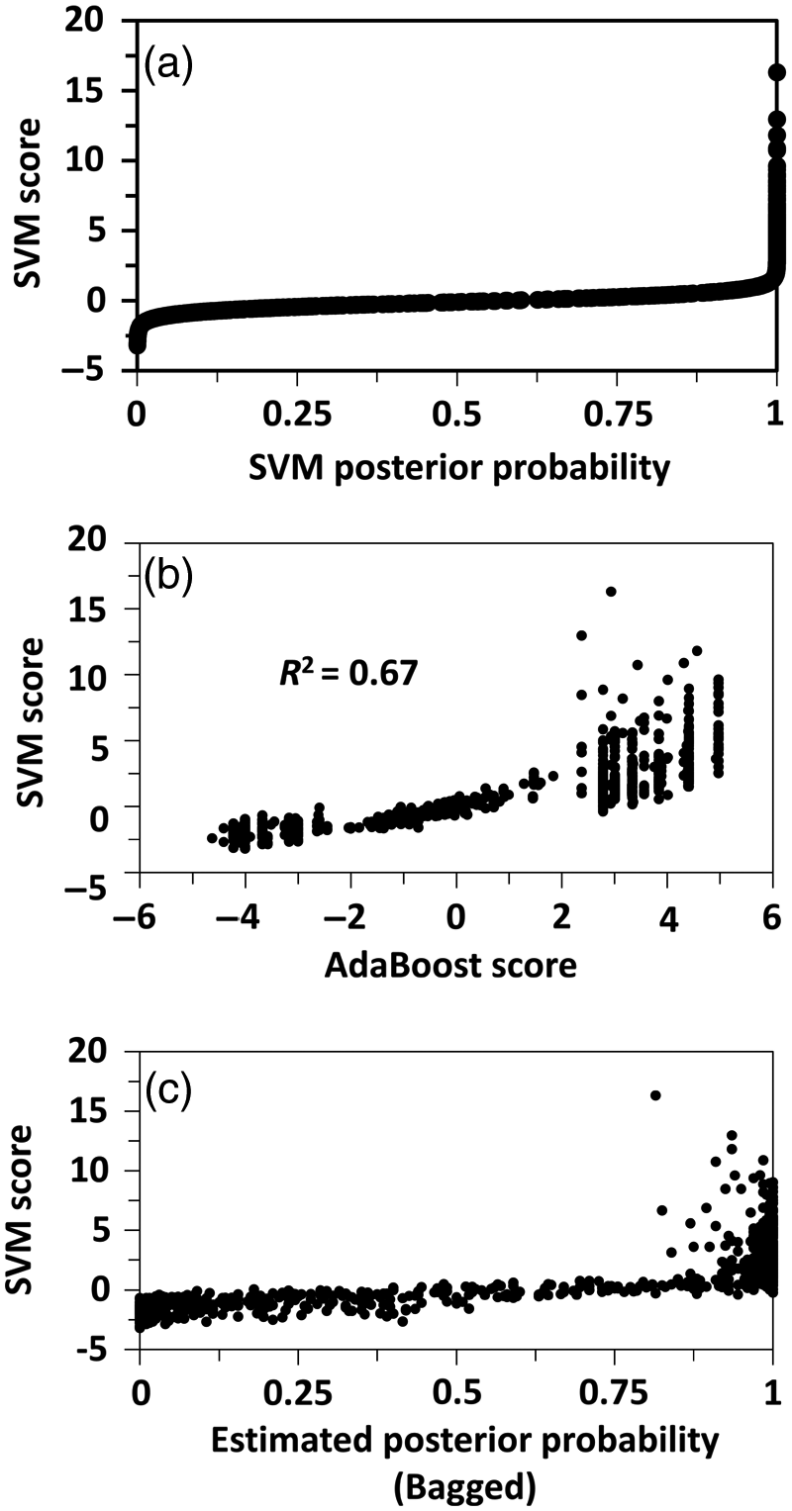
Correlation plots of linear SVM prediction scores versus (a) posterior probability scores from SVM, (b) AdaBoost prediction scores, and (c) estimated posterior probability scores from Bagged Trees for the dataset of 814 cells defined previously from GIE, HGF, MCF-7, and MDA-MB-231 cell lines.

The distributions of prediction scores from linear SVM as Euclidean distance from the classifying hyperplane [Fig. 5(a)], posterior probabilities [Fig. 5(b)], Boosted Trees [Fig. 5(c)], and Bagged Trees [Fig. 5(d)] were evaluated. Histograms of linear SVM prediction scores [Fig. 5(a), Eq. (1)] produced the most normal-appearing distributions for test data of GIE and HGF and transfer datasets of MCF-7 and MDA-MB-231 cells. Posterior probabilities from SVM [Fig. 5(b), Eq. (2)] and estimated posterior probabilities from Boosted Trees [Figs. 5(c), Eq. (3)] demonstrated excellent separation of classes, but weighted toward 0 and 1. The Boosted Trees predictions produced bimodal distributions of MCF-7 and MDA-MB-231 cell scores [Fig. 5(d), Eq. (4)]. Four scores from HGF cells were outliers and were not included in the histograms. The outliers were extremely high SVM scores more than 5.6 standard deviations away from the population mean score. Correlations between linear SVM prediction scores versus posterior probabilities [Fig. 6(a)] and versus estimated posterior probabilities from Bagged Trees [Fig. 6(b)] were highly nonlinear for low and high scores of each. The correlation between linear SVM versus Boosted Trees prediction scores [Fig. 6(c)] was linear for central scores, but nonlinear overall, with discrete levels of Boosted Trees scores favored at low and high ends of the score range.

**Fig. 7:**
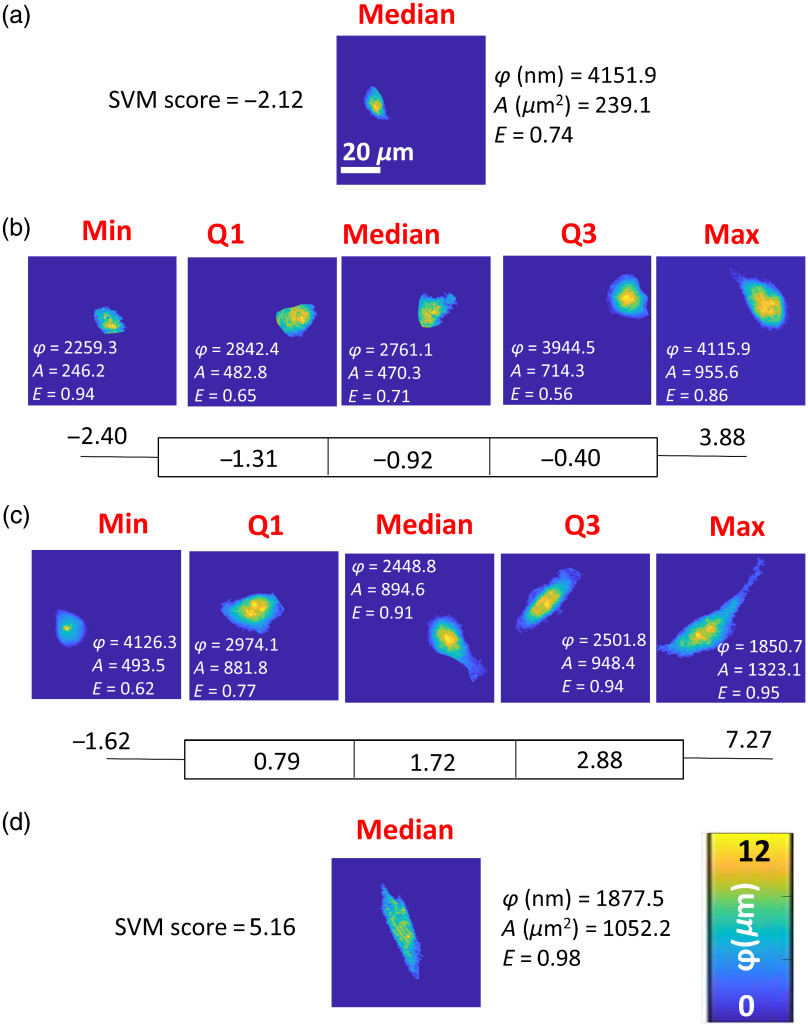
Phase maps of epithelial, mesenchymal, and breast cancer cells representing the median SVM score of normal cell line (a) GIE and (d) HGF. The minimum, first quartile, median, second quartile, and maximum SVM score for cancer cell lines of (b) MCF-7 and (c) MDA-MB-231 are shown. SVM scores were derived from a binary classification SVM model trained on GIE and HGF cells, then tested on breast cancer cells to generate weighted classification scores. Phase height (φ) in nm, area (A) in $\mu m^{2}$, and eccentricity (E) of each representative cell generated from phase maps are also listed in the figure near each cell.

**Fig. 8:**
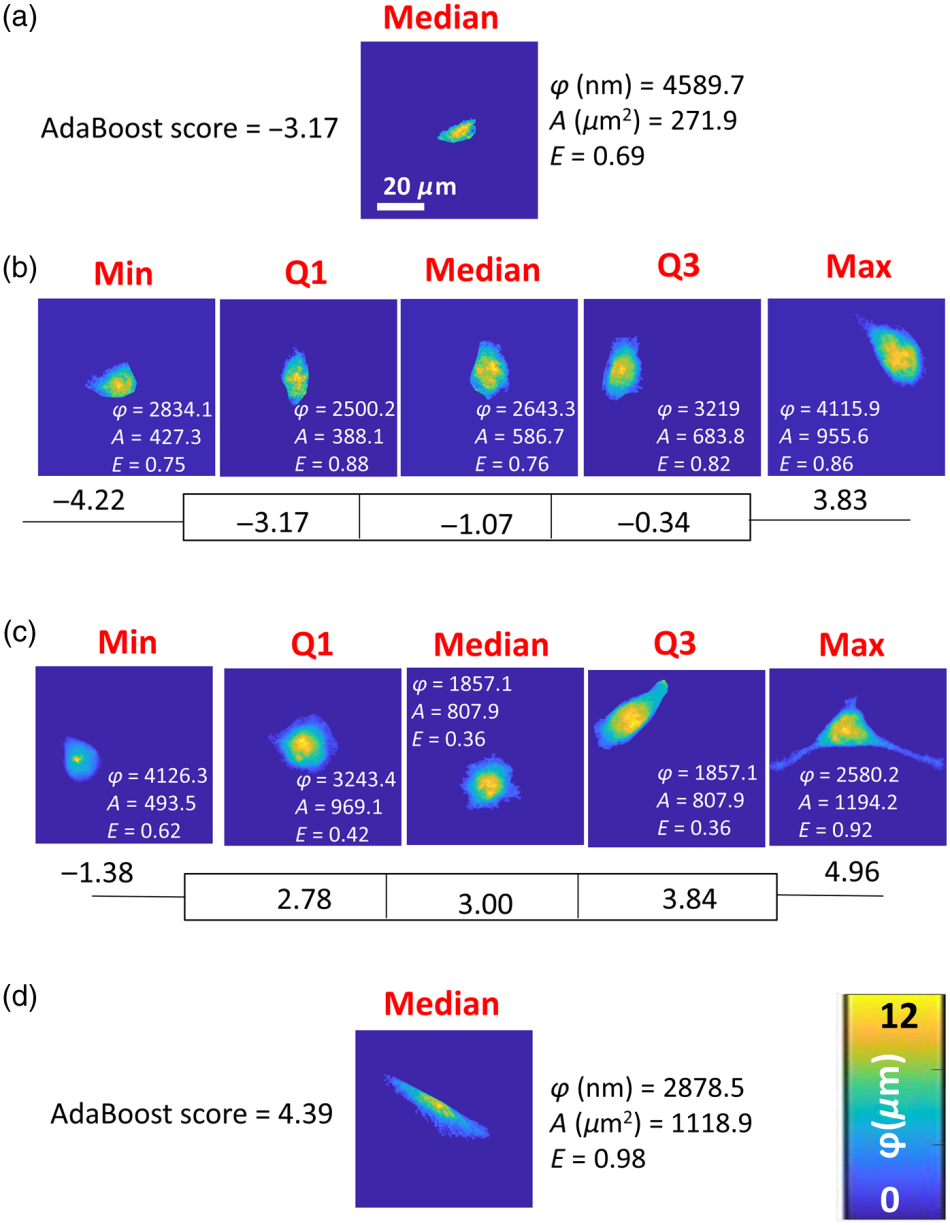
Phase maps of epithelial, mesenchymal, and breast cancer cells representing the median AdaBoost score of normal cell line (a) GIE and (d) HGF. The minimum, first quartile, median, second quartile, and maximum AdaBoost score for cancer cell lines of (b) MCF-7 and (c) MDA-MB-231 are shown. AdaBoost scores were derived from a binary classification AdaBoost model trained on GIE and HGF cells, then tested on breast cancer cells to generate weighted classification scores. Phase height (φ) in nm, area (A) in $\mu m^{2}$, and eccentricity (E) of each representative cell generated from phase maps are also listed in the figure near each cell.

Cell phase maps representing the linear SVM (Fig. 7) and Boosted Trees (Fig. 8) prediction scores closest to minima, maxima, medians, and first and third quartiles demonstrated a graded appearance between epithelial and mesenchymal phenotypes, as represented by the cells nearest the median score from GIE and HGF cells, respectively (also depicted in Figs. 7 and 8). The selected cells are for the most part different (except for the MCF-7 Max and MDA-MB-231 Min, which were the same from the two scores), but reflect a trend of more mesenchymal morphology with higher score. Other features, including phase height (nm), area ($\mu m^{2}$), and eccentricity, were also included in each representative map demonstrating each cell’s shape features. Cell phase height tended to decrease while area and eccentricity tended to increase when cells were more mesenchymal-like. Nevertheless, each geometrical feature itself did not completely correlate in rank order with the EM score derived from SVM [Eq. (1)] or from AdaBoost [Eq. (3)], for both MCF-7 [Figs. 7(b), 8(b)] and MDA-MD-231 [Figs. 7(c), 8(c)] cell lines.

## 4 Discussion

Machine learning algorithms applied to QPI of adherent cells in culture classify cell lines in a way useful for determining the functional phenotype on an EM axis. This study proposes a transfer learning approach to define a graded phenotypic classification for breast cancer cells: train a binary classifier on known epithelial and mesenchymal cells, then test on the cancer cells of unknown phenotype, defining prediction scores for each unknown cell. The algorithms producing score distributions of cancer cells most evenly distributed between epithelial and mesenchymal extremes were linear SVM and Boosted Trees (AdaBoost) scores. The SVM score, the Euclidean distance to the linear hyperplane separating epithelial and mesenchymal classes, produced normal-appearing distributions within the cancer cell lines, easily interpretable as lying along an EM continuum. The Boosted Trees score also produced a prediction score capable of being interpreted as an EM continuum, but with bimodal score distributions for the cancer cell transfer learning predictions. These prediction scores from binary classifiers serve as robust, quantitative EM scores to define unknown cells with morphologies blended in between two morphological extremes.

This proof-of-concept study has several strengths in design and analyses, but also weaknesses related to the necessarily limited dataset. Strengths include the large numbers of cells imaged (>300 per cell line) and use of MCF-7 and MDA-MB-231 cell lines, well characterized as being more epithelial and mesenchymal in nature, respectively. QPI using DHM provides high accuracy of cell morphological measurements as well as pixel-level textural details.^3^^,^^4^ One potential concern is the consistency of the scoring results if different noncancerous epithelial and mesenchymal cells were used instead of the gingival cells available for this study. The smooth histogram in Fig. 5(a) and graded appearance of cells from low to high scores in Figs. 7 and 8 increase confidence in the broad applicability of the developed scores. Still, a classifier trained on syngeneic noncancerous cells derived from the same tissue as the cancer would likely be more patient-specific. A classifier trained on data from many normal epithelial and mesenchymal cell lines would arguably be more generalizable across many patients. The bimodal nature of AdaBoost prediction scores for breast cancer cells [Fig. 5(c)] and granularity of AdaBoost scores at low and high ends of the range [Fig. 6(b)] are a weakness of the AdaBoost predictions compared to linear SVM prediction scores. This is explained by the iterative AdaBoost training algorithm that trains more learners on data that is harder to classify, i.e., it is misclassified by initial learners in the ensemble method. This results in a finer resolution of scores in the middle of the score range, which are the harder to predict cases. The proposed EM score requires validation with additional cancer cells of different epithelial and mesenchymal morphologies from various breast cancer subtypes.^59^ Despite these limitations of the current training and test datasets, the transfer learning method proposed here quantitatively sorts individual cells along a putative morphological axis that produces well-ranked cells by visual inspection, a feat not reproducible using any single geometrical feature to rank the cells, including thickness, area, and eccentricity (Figs. 7 and 8).

Morphological evaluation using an EM score as proposed here could address a major issue in histology-based diagnostics derived from cell-to-cell heterogeneity. Such heterogeneity, especially in the absence of specific molecular biomarkers, makes risk stratification, diagnosis, and selection of treatment regimens less accurate.^60^ Rapid morphological classification of biopsied cancer cell populations in slide sections using DHM could provide a first-order evaluation of cancer heterogeneity, especially if morphology were linked to clinical behavior. Interestingly, intravital microscopy suggests that cancer cells undergo EM transition to enable metastasis but can rapidly revert to epithelial phenotypes once in the metastatic site, a process termed EM plasticity.^61^ An EM score applied to individual cells might, therefore, be useful as an indicator of recent, concurrent, or impending metastasis within the sampled cancer cell population. Such a score could also track responses of the two cancer subpopulations to combined epithelial- and mesenchymal-directed therapies, such as inhibition of Wingless/Integrated (*Wnt*) and Yes-associated protein (*YAP*) signaling.^62^

Quantitative sorting of adherent cells based on morphology is of potential utility in phenotypic screening and basic studies linking gene expression to phenotype and functional behavior. In phenotypic screening, DHM followed by assignment of a machine learning prediction score to individual cells would allow the detection of subtle morphological shifts in response to various treatments,^63^ a task of increasing importance in drug discovery.^64^ In this study, two breast cancer cell lines, MCF-7 and MDA-MB-231, were scored on an EM axis by linear SVM, consistent with their morphological appearance. These cell lines are well known for appearing with clustered epithelial-type and single, mesenchymal-type morphologies, respectively. Mesenchymal gene expression in MDA-MB-231 cells, including N-Cadherin, Snail, Slug, ZEB1 and 2, and Yes-associated protein 1 (YAP1), were downregulated after lentiviral insertion of E-cadherin, a marker of epithelial cells, which shifted the morphology of MDA-MB-231 cells to a more rounded, clustered epithelial type.^62^ Similarly, MCF-7 cells made to express Snail, a transcription factor typical of mesenchymal cells, become less round and experience an upregulation of mesenchymal-related genes and downregulation of epithelial-related genes.^65^ Such basic studies have potential impact in defining the roles of epithelial and mesenchymal phenotypes in cancer behavior, leading to a better understanding of phenotypic transitions and plasticity in cancer. An EM score would aid such efforts by establishing the magnitude of phenotypic shifts with a given treatment.

An EM score has utility in interpreting qualitative morphological assignments. For example, breast cancer cells in three-dimensional views have been classified as “stellate,” “grape-like,” “mass,” or “round,” and unique gene expression profiles are linked to these classes.^40^ A single, unified EM score applied to each of these classes may correlate with the expression of multiple key genes, linking morphology to gene expression profiles on a quantitative basis. One exciting future development of such an approach would be to determine the sensitivity of the EM score to differential expression of individual genes, something best achieved by direct comparison of parental and genetic knockout cell lines. There is some evidence that qualitative morphological classes do not correspond to invasiveness in all cases.^43^ This finding is consistent with at least a subset of genes being responsible for invasiveness but not aggregate morphology, a hypothesis which is testable through sequential genetic knockout, EM scoring, and assessment of invasiveness *in vitro*. QPI of cancer cells in functional assays combined with classification scores such as the proposed EM score could aid such studies. In single-cell studies, phase images of cells of interest could guide laser-capture microdissection to link observed behavior, morphology, and gene expression at a single cell level. Indeed, advanced machine learning techniques, including deep learning,^29^ have recently been applied to isolate cell subpopulations based on unique phase features^6^ and other phenotypic differences,^66^^,^^37^ including metastatic versus primary cancer^67^ and different types of nonactivated lymphocytes.^68^ The phase/morphology score concept described here could be applied to support decision-making in intelligent cell sorting systems, such as flow cytometry with QPI,^69^^,^^34^ to partition cells from a heterogeneous population into distinct morphological groups.^70^

The proposed technique to generate EM scores offers greater robustness, adaptability, and flexibility than qualitative or single-parameter morphological characterization, but requires some interpretation. First, the SVM derived score is <0 for all cells classified as epithelial and >0 for all cells classified as mesenchymal, whatever their true origin. Robustness of the score derives from drawing upon multiple (six) PCs for classification and is demonstrated in Figs. 7 and 8 by the biophysical/geometrical parameters of phase height, area, and eccentricity, which are not in rank order for rank-ordered EM scores within the cancer cell lines. Whereas any single parameter suffers from heterogeneity from cell to cell or may lose sensitivity to some cells or cell responses,^39^ multiple training features for machine learning classification regularly achieve higher performance than single-feature classification.^18^ Score adaptability stems from flexibility in defining the training dataset—different cell lines or primary cells could be used—as well as the cells to be scored by transfer learning. For example, the algorithm applied here to breast cancer cells could equally be applied to any cell type with a mixture of epithelial and mesenchymal qualities, such as cells undergoing epithelial-to-mesenchymal or mesenchymal-to-epithelial transition.^71^

## 5 Conclusion

This study proposes morphological scoring to sort unknown cells along a recognizable morphological axis using quantitative phase signatures and machine learning. As an example related to cancer cell phenotypes, phase features from well-characterized epithelial and mesenchymal cell lines were trained using SVM, producing a linear EM score applicable to cancer cells. This proposed morphological score has various future applications in characterizing individual cancer cells of unknown lineage and/or phenotype, and the general approach is applicable in comparing any cells to the morphologies of well-known, well-characterized cell lines.
