# Supplementary material for: Quantitative scoring of epithelial and mesenchymal qualities of cancer cells using machine learning and quantitative phase imaging
Source: J Biomed Opt. 2020 Feb 18;25(2):026002. doi: 10.1117/1.JBO.25.2.026002 (PMC7026523; doi:10.1117/1.JBO.25.2.026002)
Supplement: Supplementary file 1 [file JBO_025_026002_SD001.docx]

**SUPPLEMENTAL** **DATA**

**Table S1. List of 17 cell parameters generated from DHM segmentation written Matlab code**

| **μ** | CellMean | Average phase height | 32-bit full, in nm | $\mu=\frac{1}{N}\sum_{i=1}^{N} x_{i}$ |
| --- | --- | --- | --- | --- |
| **SD** | CellStd | Phase height standard deviation | 32-bit full, in nm | $SD=\sqrt{\frac{1}{N-1}\sum_{i=1}^{N} {\vert A_{i-}\mu\vert}^{2}}$ |
| **ku** | Kurt | Phase height kurtosis | 32-bit full | $k=\frac{E{(x-\mu)}^{4}}{\sigma^{4}}$ |
| **sk** | Skew | Phase height skewness | 32-bit full | $sk=\frac{E{(x-\mu)}^{3}}{\sigma^{3}}$ |
| **Ar** | Stats.Area | Segmented cell area | Units of pixels | -- |
| **Ecc** | Stats.Eccentricity | Segmented cell fit ellipse eccentricity | Units of pixels | -- |
| **Per** | Stats.Perimeter | Segmented cell perimeter | Units of pixels | -- |
| **Co** | Stats2.Contrast | 2^nd^ order texture parameter | 8-bit scaled | $\sum_{i,j} \left\vert i-j \right\vert^{2}p(i,j)$ |
| **Cor** | Stats2.Correlation | 2^nd^ order texture parameter | 8-bit scaled | $\sum_{i,j} \frac{\left( i-\mu i \right)\left( j-\mu j \right)p(i,j)}{\sigma_{i}\sigma_{j}}$ |
| **En** | Stats2.Energy | 2^nd^ order texture parameter | 8-bit scaled | $\sum_{i,j} p{(i,j)}^{2}$ |
| **Hm** | Stats2.Homogeneity | 2^nd^ order texture parameter | 8-bit scaled | $\sum_{i,j} \frac{p(i,j)}{1+\vert i-j\vert}$ |
| **µ_n_** | Central region mean | Average central region phase height | 32-bit full, in nm | See Cell Mean |
| **µ_nm_** | Central region maximum mean | Maximum phase height, central region | 32-bit full, in nm | -- |
| **a** | Central region area | Segmented area, central region | Units of pixels | -- |
| **sd** | Central region Std | Phase height standard deviation | 32-bit full, in nm | See Cell Standard Deviation |
| **k_n_** | Central region kurtosis | Phase height kurtosis | 32-bit full | See kurtosis |
| **sk_n_** | Central region skew | Phase height skewness | 32-bit full | See skew |
